# SOD1 and DJ-1 Converge at Nrf2 Pathway: A Clue for Antioxidant Therapeutic Potential in Neurodegeneration

**DOI:** 10.1155/2013/836760

**Published:** 2013-07-28

**Authors:** Pamela Milani, Giulia Ambrosi, Omar Gammoh, Fabio Blandini, Cristina Cereda

**Affiliations:** ^1^Laboratory of Experimental Neurobiology, National Neurological Institute C. Mondino, IRCCS, Via Mondino 2, 27100 Pavia, Italy; ^2^Department of Public Health, Neuroscience, Experimental and Forensic Medicine, University of Pavia, Via Ferrata 9, 27100 Pavia, Italy; ^3^Laboratory of Functional Neurochemistry, Center for Research in Neurodegenerative Diseases, National Neurological Institute C. Mondino, IRCCS, Via Mondino 2, 27100 Pavia, Italy; ^4^Department of Brain and Behavioral Sciences, University of Pavia, Via Ferrata 9, 27100 Pavia, Italy; ^5^Faculty of Health Sciences, American University of Madaba, Madaba, Jordan

## Abstract

Neurodegenerative diseases share diverse pathological features and among these oxidative stress (OS) plays a leading role. Impaired activity and reduced expression of antioxidant proteins have been reported as common events in several aging-associated disorders. In this review paper, we first provide an overview of the involvement of reactive oxygen species- (ROS-) induced oxidative damage in Parkinson's disease (PD) and amyotrophic lateral sclerosis (ALS). Subsequently, we focus on DJ-1 and SOD1 proteins, which are involved in PD and ALS and also exert a prominent role in the interaction between redox homeostasis and neurodegeneration. Interestingly, recent studies demonstrated that DJ-1 and SOD1 are both tightly connected with Nrf2 protein, a transcriptional factor and master regulator of the expression of many antioxidant/detoxification genes. Nrf2 is emerging as a key neuroprotective protein in neurodegenerative diseases, since it helps neuronal cells to cope with toxic insults and OS. We herein summarize the recent literature providing a detailed picture of the promising therapeutic efficacy of Nrf2 natural and synthetic inducers as disease-modifying molecules for the treatment of neurodegenerative diseases.

## 1. Introduction

Oxidative stress (OS) is a crucial player in several diseases, including age-dependent neurodegenerative disorders such as Parkinson's disease (PD) and amyotrophic lateral sclerosis (ALS). OS accumulation in postmitotic neurons during aging represents a phenomenon of significant relevance since it can trigger a vicious cycle of intracellular damages, ultimately resulting in neuronal cell death. 

The involvement of OS in several neurodegenerative conditions has been demonstrated by the identification of pathological mutations in genes prominently featuring in defensive pathways as well as OS markers in patients' samples (as reviewed in [1–4]). Nevertheless, in many cases it is not clear whether this kind of stress is a primary cause or rather an ongoing downstream event associated with the progression of the neurodegenerative process.

OS is typically defined as the imbalance between the production of reactive oxygen species (ROS) and the efficient removal of these species by cellular defensive mechanisms, which include both enzymatic scavengers (e.g., superoxide dismutases, catalase, glutathione peroxidase, glutathione reductases, and peroxiredoxins) and low-molecular-weight reductants (e.g., vitamin E, glutathione, and ascorbate). Mitochondria use approximately the 85–90% of total oxygen, thus representing the major site of oxygen consumption as well as a primary and continuous source of cellular ROS. ROS such as superoxide (O^−2^) and hydrogen peroxide (H_2_O_2_) principally originate as by-products of aerobic metabolism, due to electron “leakage” from the mitochondrial respiratory chain during oxidative phosphorylation with the consequent incomplete reduction of molecular oxygen. A more limited percentage of intracellular ROS arise from the activity of oxidative enzymes, including the cytochrome P450 system associated with the endoplasmic reticulum, the cytoplasmatic xanthine oxidase, the membrane enzyme NADPH oxidase [5], and p66Shc, an important regulator of intracellular redox balance, mitochondrial permeability, and apoptosis [6]. Superoxide itself is not highly dangerous; nevertheless it can rapidly react with the mild oxidant Nitric Oxide (NO), produced by the nitric oxide synthase (NOS), to generate the more harmful peroxynitrite (ONOO^−^) [7, 8]. Likewise, H_2_O_2_ is a weak oxidant but it gradually decomposes to generate the hydroxyl radical (^•^OH), one of the most toxic-free radicals in biological systems. Both ONOO^−^ and ^•^OH impair the function of biomolecules by affecting several targets inside the cell. Specifically, ROS attack the backbone and the side chains of proteins causing the formation of carbonyl groups and methionine sulfoxide and often determining protein misfolding and aggregation. In addition, they attack nucleic acids, leading to DNA single- and double-strand breaks, DNA-protein crosslinks, and/or modification of purine and pyrimidine bases, and to oxidative modification in both protein-coding RNAs and noncoding RNAs. Furthermore, ROS cause lipid peroxidation, a complex phenomenon involving the interaction between unstable free radicals and polyunsaturated fatty acids, yielding highly reactive products, such as malondialdehyde, 4-hydroxy-2-trans-nonenal (HNE), acrolein, and thiobarbituric acid reactive substances (TBARS) [9]. In synthesis, OS causes a cascade of damaging processes eventually leading to cell death. 

Although all the aerobic cells are subjected to oxidative damage, neurons are particularly vulnerable to the injuring effects of by-products derived from the oxidative metabolism. This susceptibility can be ascribed to their high metabolic requirements and oxygen demand combined with a relatively low expression of antioxidant proteins, in particular catalase (as reviewed in [1, 10]), and their limited regenerative capacity. 

While an exaggerate production of ROS is typically associated with broad deleterious effects for neuronal cell functions and viability, increasing body of evidence is demonstrating that changes in redox environment, including generation of oxidants, also exert crucial roles in regulating specific signalling events. In particular, ROS have been shown to be involved in kinase cascade activation [11], calcium mobilization and signalling [12, 13], fine-tuned control of redox-sensitive gene expression [14, 15], and, more recently, in neural stem cell differentiation [16] and neurogenesis [17]. 

Consequently, a better understanding of ROS involvement in determining the fate of neuronal cells may yield clues to the pathogenesis of neurodegenerative diseases and may offer the possibility to pharmacologically manipulate intracellular molecular pathways, redox-sensitive transcriptional events, and antioxidant systems as promising neuroprotective therapies.

## 2. Parkinson's Disease

Parkinson's disease affects more than 1% of the population over 60 years of age and is the second most common neurodegenerative disorder after Alzheimer's disease (AD) [18]. The majority of cases (90%) are sporadic, while about 10% show monogenic inheritance [19]. 

PD is caused by the degeneration of dopaminergic neurons within the substantia nigra pars compacta (SNc) and although there is still no clear explanation for the intrinsic vulnerability of these neurons, it is known that they are more prone and susceptible to OS. In fact, several prooxidant factors constitutively challenge SNc dopaminergic neurons: in particular pacemaking activity, sustained calcium buffering, dopamine self-oxidation, and iron oxidation [20]. PD pathogenesis is indeed complex and multifactorial and selective vulnerability of SNc dopaminergic neurons can be further stressed by converging pathogenic mechanisms that include a predisposing genetic background, exposure to environmental neurotoxins, defective proteolytic systems, and impaired mitochondrial integrity and function [21]. In particular, mitochondrial defects lead to impaired energy and ROS production and therefore to altered bioenergetic and redox balance.

Consistent evidence from both genetic and epidemiological studies shows that disrupted mitochondrial integrity and OS play a pivotal role in PD pathogenesis and disease progression. Genes such as *PARK2*, *PARK6*, and *PARK7* encoding, respectively, for Parkin, PINK1, and DJ-1 are associated with early-onset familial forms of PD and mutations in all of those genes affect mitochondrial health and function thereby causing neuronal death [22]. Epidemiological data and animal models demonstrate that environmental toxins and pesticides that inhibit mitochondrial complex I, such as 1-methyl-4-phenyl-1,2,3,6-tetrahydropyridine (MPTP) and rotenone, are responsible for bioenergetic crisis and, most importantly, increased ROS production and OS, eventually causing loss of dopaminergic neurons in the SNc [23]. 

Furthermore, markers of OS are typically found in brain biopsies, peripheral cells, and biological fluids derived from patients with PD, indicating that indeed OS is a key factor in PD pathogenesis. In postmortem brains derived from PD patients increased accumulation of both carbonylated proteins and markers of lipid peroxidation such as TBARS were detected [24]. Similarly, markers of lipid peroxidation were increased in plasma and cerebrospinal fluid (CSF) derived from PD patients as compared to controls [25]. Nonetheless, 8-hydroxy-2′-deoxyguanosine (8-OHdG), an indicator of nucleic acid oxidation, in particular of nuclear and mitochondrial DNA damage, was increased in CSF of patients with PD, together with augmented levels of oxidized Coenzyme Q10 [26]. PD has also been associated with alterations in the expression of antioxidant molecules such as glutathione and antioxidant enzymes. It was shown that oxidized glutathione is significantly higher in blood cells from PD patients as compared to controls and concentrations of other antioxidant molecules and catalase activity are decreased [27]. Furthermore, several studies have shown that activation of antioxidant genes expression, in particular those under the control of the Nrf2/ARE system (i.e., NQO1 and GST; see Section 5.1), has neuroprotective effects in different models of PD [28, 29]. 

Finally, OS can also drive PD progression through the activation of excitotoxic phenomena and neuroinflammatory processes. Excitotoxicity is due to the hyperactivation of glutamatergic receptors, in particular *N*-methyl-D-aspartate (NMDA). Activation of these receptors leads to intracellular calcium overload which triggers ROS formation and the release of proapoptotic factors [30]. The activation of neuroinflammatory processes is mediated by glial cells, astrocytes, and more severely microglia. After a primary neuronal insult, microglia remains persistently activated and contributes to the release of free radicals which exacerbate the neurodegenerative process and accelerate its progression [31].

## 3. Amyotrophic Lateral Sclerosis (ALS)

ALS is a rare adult-onset neurodegenerative disease characterized by the selective degeneration of motor neurons in the motor cortex, brainstem, and spinal cord. Most of the cases (90%) are sporadic (SALS), while the remainder presents a family history (FALS). 

Although the exact cause of ALS is still unknown, a major step forward in the understanding of the pathogenetic events involved in ALS was provided in 1993 by the observation that mutations in the gene coding for the antioxidant enzyme Cu/Zn superoxide dismutase (SOD1) are carried by the 15–20% of FALS patients [32]. 

Growing evidence suggests that ALS is a complex and multifactorial disease characterized by the involvement of several interconnected pathogenic events, such as OS, mitochondrial dysfunction, inflammation, glutamate excitotoxicity, protein misfolding and aggregation, aberrant RNA metabolism, and altered gene expression [33–37]. In particular, OS is one of the most detrimental contributors of disease onset and progression. In fact, several distinctive oxidation markers have been observed in both nervous and peripheral tissues in SALS and FALS patients [38–40]. Elevated protein carbonyl and 3-nitrotyrosine levels have been detected in spinal cord and motor cortex from SALS and FALS patients, particularly in large ventral motor neurons [38, 41–43]. Lipid oxidation has also been identified in motor neurons, astrocytes, and microglia of SALS patients compared to control individuals [44, 45]. Elevated levels of HNE have been detected also in CSF [46] and in sera [47] from ALS patients. Additionally, mitochondrial defects have been reported as a major hallmark in motor neuron degeneration in ALS [48, 49]. These dysfunctions are tightly interrelated with OS cascades, activating overlapping molecular pathways in a vicious cycle of harmful events. Specifically, alterations in mitochondrial morphology and biochemistry have been extensively detected in postmortem tissues [50] and in lymphocytes [51] from SALS patients, in SOD1 transgenic mice and cellular models [52]. Dynamic and morphological abnormalities, such as swelling and vacuolization, along with metabolic deficits in the activities of the respiratory chain complexes have been also described both in SALS and FALS patients [53]. These defects lead to both bioenergetic failure and increased ROS generation.

Notably, impairment in defensive mechanisms has also been revealed in ALS, including downregulation of members of glutathione S-transferase family [54, 55], peroxiredoxins [56], and, in particular, the transcriptional factor Nrf2 [57–60].

## 4. DJ-1 and SOD1 Involvement in Oxidative Stress and Neurodegeneration

### 4.1. DJ-1

DJ-1 is a ubiquitously expressed protein encoded by *PARK7* gene, initially identified as an oncogene functionally associated with cancer and male infertility [61, 62]. Mutations in *PARK7* gene, leading to loss of function of the protein, were later associated with early-onset recessive forms of PD [63].

**Figure 1:**
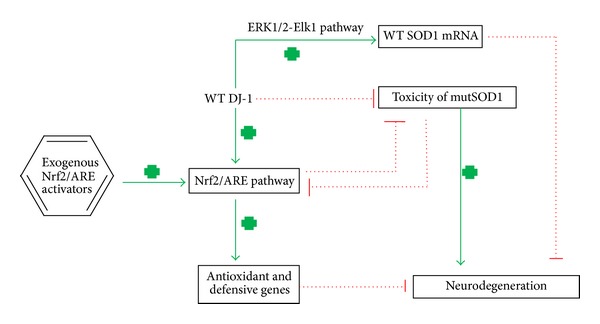
WT DJ-1 directly protects from PD and activates ERK1/2-ELK1 pathway thus upregulating WT-SOD1 expression. Also WT DJ-1 activates Nrf2/ARE pathway hence inducing antioxidant genes. Furthermore, both WT DJ-1 and Nrf2/ARE inhibit the toxicity of mutSOD1 that causes ALS, and vice versa, Nrf2/ARE pathway is inhibited by mutSOD1. Finally, exogenous Nrf2/ARE activators represent a powerful tool in the induction of antioxidant and defensive genes.

DJ-1 is a small homodimeric protein composed of two subunits of 189 amino acid residues with a molecular weight of approximately 20 kDa. The crystal structure of this protein was independently solved by several groups [64–67], providing a helpful framework for a better understanding of its possible molecular activities. Specifically, it has a flavodoxin-like core fold, composed of *α*-helical layers sandwiching a six-strand parallel *β*-sheet. PD-associated mutations lead to diverse levels of protein folding defects or structural perturbations with consequent functional alterations of the protein [68]. DJ-1 is mainly localized in the cytoplasm, but under OS the protein can be recruited either to the mitochondria or to the nucleus [69–71]. It has been reported that after oxidative insult, DJ-1 translocates to the mitochondria within 3 hours and to the nucleus after 12 [72], suggesting that timing after oxidative challenge is essential to determine the subcellular compartment where the protein is active. Moreover, DJ-1 stimulates the cytoprotective pathway mediated by extracellular-signal-regulated kinase (ERK1/2) and its substrate ETS domain-containing protein (Elk1) (Figure 1). It was demonstrated by Gu and colleagues in two different cell lines that overexpression of wild-type DJ-1 increases ERK1/2 phosphorylation leading to Elk1 activation, thereby decreasing cell susceptibility to H_2_O_2_ and increasing cell viability [73].

Although the exact function of DJ-1 remains unclear, increasing studies have revealed that the protein is involved in various biological processes, in particular control of ROS levels and OS-induced apoptosis. DJ-1 responds to increased ROS levels by oxidizing itself, in particular at Cystine 106 (C106). Site-directed mutagenesis of C106, performed in both cellular and animal models, established a clear role for C106 oxidation in DJ-1 function, since mutations interfered with the antioxidant, antiapoptotic, and eventually neuroprotective effect of the protein [74–76]. 

Through self-oxidation, DJ-1 acts as a sensor of cellular redox state [77] and also as a redox-activated peroxidase with peroxiredoxin-like activity [74]. DJ-1 is therefore a signalling molecule responsive to cellular redox state which exerts antioxidant functions because of its capability of buffering oxidized molecules not only by physically interacting with them at C106, but also by inducing the expression of antioxidant defences [78, 79]. Finally, according to its oxidative state, DJ-1 counteracts the induction of apoptotic mechanisms [80] and protects neuron viability by acting as a chaperone [81] and autophagy modulator [82].

Therefore, the essential role of DJ-1 is to protect cells from apoptotic death triggered by harmful levels of ROS by immediately protecting mitochondrial integrity and, after sustained oxidative challenge, by modulating transcription of antioxidant genes.

### 4.2. SOD1

One of the primary cellular defensive systems for oxidative insults is the antioxidant enzyme SOD1. It is one of the three human superoxide dismutases identified and characterized in mammals: copper-zinc superoxide dismutase (Cu/ZnSOD or SOD1), manganese superoxide dismutase (MnSOD or SOD2), and extracellular superoxide dismutase (ECSOD or SOD3). It is a 32 kDa homodimer of a 153-residue polypeptide with one copper- and one zinc-binding site per subunit. Specifically, each monomer possesses a *β*-barrel motif and two large functionally important loops, called the electrostatic and zinc loops, which encase the metal-binding region. It catalyzes the reaction of superoxide anion (O^2−^) into molecular oxygen (O_2_) and hydrogen peroxide (H_2_O_2_) at a bound copper ion [83]. The intracellular concentration of SOD1 is high (ranging from 10 to 100 *μ*M) [84] counting for 1% of the total protein content in central nervous system (CNS). The protein is localized not only in the cytoplasm but also in nucleus, lysosomes, peroxisomes, and mitochondrial intermembrane spaces in eukaryotic cells [84, 85].

As mentioned before, the first evidence of the involvement of SOD1 in familial ALS was provided by Rosen and coworkers [32]; currently, more than 150 different mutations distributed throughout the 153-amino acid SOD1 polypeptide have been linked to ALS. These are predominantly single amino acid substitutions although deletions, insertions, and C-terminal truncations also occur. Initial hypothesis regarding its involvement in mediating motor neuron degeneration suggested that mutSOD1 displays reduced activity, promoting accumulation of toxic superoxide radicals [32]. However, several molecular and functional studies performed in animal and cellular models showed that SOD1 pathogenic variants cause FALS by gain rather than loss of function. In this regard, Pesaresi and coworkers [86] demonstrated that mutSOD1 activates p66Shc, which is known to affect mitochondrial function and mitochondria-dependent oxidative balance. Furthermore p66Shc activation inhibits the activity of Rac1, an intracellular transducer which mediates several pathways associated with gene expression regulation, cell proliferation, and cytoskeleton organization [87], thereby causing further OS. Interestingly, another group showed that SOD1 creates a self-regulated redox cycle by directly interacting with Rac1 and orchestrating NADPH oxidase-dependent superoxide production. The study therefore suggests that some mutations in SOD1 might contribute to a gain of function of the protein through the disruption of such redox control [88]. Other hypotheses concerning the role of SOD1 in FALS postulate that mutSOD1 acquires toxic properties that are independent of its normal physiological function. The investigation on the toxic function acquired by mutSOD1 led to the proposal of two main hypotheses [33]. In the aberrant redox chemistry model, mutSOD1 is unstable and through aberrant chemistry interacts with nonconventional substrates causing ROS overproduction. In the protein toxicity model, unstable, misfolded SOD1 aggregates into cytoplasmic inclusion bodies, sequestering proteins crucial for cellular processes. These two hypotheses, however, are not mutually exclusive. Indeed, it has been shown that oxidation of selected histidine residues that bind metals in the active site mediates SOD1 aggregation [89].

### 4.3. DJ-1 Modulates MutSOD1 Motor Neuron Degeneration Processes in ALS

Recent observations described a connection between DJ-1 and mutSOD1 in ALS (Figure 1). The first evidence about such connection was described by Lev and coworkers [90], who detected increased DJ-1 mRNA and protein levels in the brains and spinal cords of SOD1-G93A transgenic mice, a widely employed model of ALS. The upregulation was detectable starting from the very early stages of disease progression. In addition, enhanced levels of DJ-1 acidic isoforms were found, indicating that more oxidized DJ-1 amounts were present in the CNS of transgenic mice. In 2010, Yamashita and collaborators [91] proved, both *in vitro* and *in vivo*, the existence of direct association between DJ-1 and SOD1: the two proteins interacted in GST pull-down assays and formed complexes that colocalized in mice primary motor neuron culture. A better colocalization was obtained between DJ-1 and mutSOD1 rather than WT-SOD1. Notably, the overexpression of exogenous DJ-1 in stably mutSOD1-expressing cells reduced cell toxicity and OS markers as compared to cells expressing control vector protein. These data strongly suggest that the described interaction plays a protective role, although further investigation is required to shed light on the functional events activated by DJ-1. Indeed, the exact molecular mechanisms by which DJ-1 accomplishes its defensive function(s) in ALS are still largely unknown. 

## 5. Nrf2/ARE Pathway: Relationship with DJ-1 and SOD1 Proteins

### 5.1. Nrf2 Pathway

NF-E2-related factor 2 (Nrf2) belongs to the Cap'n'collar (Cnc) transcription factor family and is considered the “master regulator” of the antioxidant response since it modulates the expression and the coordinated induction of an array of defensive genes encoding phase II detoxifying enzymes and antioxidant proteins, such as NAD(P)H: quinine oxidoreductases (NQOs), heme oxygenase-1 (HO-1), the glutathione S-transferase (GST) family, multidrug resistance-associated proteins (Mrps), the UDP-glucuronosyltransferase (UGT) family, ferritin proteins, cyclooxygenase-2 (COX-2), and inducible nitric oxide synthase (iNOS) [60, 92]. Nrf2 is a very unstable protein, typically present in association with its negative regulator Kelch-like ECH-associated protein 1 (Keap1), which acts as a molecular sensor of cellular redox homeostasis disturbance. Under basal condition, Keap1 retains Nrf2 in the cytoplasm, linking this transcriptional factor to the actin cytoskeleton and driving its degradation. Specifically, Keap1 acts as a linker protein between Nrf2 and the Cul3-based E3-ubiquitin ligase complex, promoting Nrf2 ubiquitination and consequent degradation by the 26S proteasome [93, 94].

This quenching interaction between the two proteins is a dynamic process controlled by specific intracellular cascades that allow for a fine-tuned regulation of inducible expression of Nrf2 target genes under OS or after exposure to toxic electrophiles. In fact, activation of Nrf2 requires its cytosolic stabilization via oxidative modification of distinct Keap1 cysteine residues and/or Keap1 ubiquitination and proteasomal degradation. It has been largely demonstrated that also Nrf2 phosphorylation facilitates its dissociation from Keap1. Therefore, several signaling pathways, such as the activation of mitogen-activated protein kinase (MAPK) cascade, phosphatidylinositol 3-kinase (PI3K), and protein kinase C (PKC), favour Nrf2 detachment from its repressors and the consequent translocation to the nucleus. In the nuclear compartment Nrf2 forms a heterodimer with its partner small Maf and binds specific *cis*-acting antioxidant response element (ARE) sequences, ultimately transactivating a battery of highly inducible cytoprotective genes thus allowing cell to efficiently cope with endogenous stress and exogenous toxicants [95]. Nrf2 has also been shown to modulate the transcription of genes promoting mitochondrial biogenesis, such as mitochondrial transcription factors (TFAM) [96], and consequently to be directly involved in mitochondrial maintenance.

Considering the pivotal defensive role exerted by the Nrf2/ARE pathway, it is evident that the dysregulation of Nrf2-regulated genes offers a logical explanation for the direct and indirect association between OS and several neurodegenerative conditions.

### 5.2. DJ-1 and Nrf2 Pathway

DJ-1 is a redox-sensitive protein which triggers activation of antioxidant defences in particular through Nrf2/ARE system (Figure 1). The first evidence of a connection between DJ-1 and Nrf2 was reported by Clements and collaborators who showed in different cellular models that DJ-1 affects the stability and the transcriptional functions of Nrf2. In particular they found that DJ-1 stabilizes Nrf2 by interfering with its ubiquitination and facilitates Nrf2 translocation to the nucleus by preventing the binding with Keap1 [97]. Similar results from another group confirmed the role of DJ-1 in controlling Nrf2 turnover in both cellular and animal models [98]. A very recent study showed that mutations in *PARK7* gene and protein loss of function significantly reduce thioredoxin1 (trx1) expression. Thioredoxin1 is a disulfide oxidoreductase, whose transcription is under the control of a promoter containing typical ARE sequence and is therefore expressed after Nrf2 activation. According to the authors, these findings further stress the association between DJ-1 and Nrf2 and support the hypothesis that DJ-1 action against OS in the nucleus is carried out by Nrf2 [79]. Nonetheless, Nrf2/ARE pathway activation has recently obtained growing attention and interest as a major target to develop new disease-modifying, neuroprotective strategies in PD [99].

### 5.3. SOD1 and Nrf2

The first evidence of a relationship between SOD1 and Nrf2 was reported by Kirby and coworkers [57], who showed by microarray analysis that the presence of mutSOD1 (G93A) in mouse motor neuron-like hybrid cell line NSC34 caused a reduction in Nrf2 mRNA expression as well as a global downregulation of a battery of Nrf2 target genes (Figure 1). Notably, also Nrf2 mRNA expression is reduced as compared to cells transfected with WT-SOD1. Several further studies attempted to shed light on Nrf2 signalling cascade in models for SOD1-associated FALS. Diminished Nrf2 levels have been observed in embryonic motor neuron cultures from SOD1-G93A transgenic mice. These cells were more susceptible to apoptosis caused by exposure to nerve growth factor (NGF) [100]. Opposed to these studies carried out in motor neurons, Kraft and collaborators reported a significant activation of Nrf2 in distal muscles of mutSOD1 mice during the early stages of the pathology. Nrf2-ARE activation appeared to later propagate in a retrograde manner also along the motor pathway during disease progression and was interpreted as a reactive attempt to counteract broad pathogenic signalling cascades induced by mutSOD1 toxicity [101].

Although the literature data present some contrasts and gaps on the molecular details underlying mutSOD1-Nrf2 interaction, these findings suggest that the toxic gain of function of mutSOD1 may lead to a perturbation of Nrf2 pathway activation.

On the other hand, though, pharmacological treatment aiming at activating Nrf2 pathway might counteract the negative effect exerted by mutSOD1 and might represent a promising therapeutic perspective for ALS. In support of this hypothesis, a neuroprotective role played by Nrf2 pathway activation in mutSOD1-associated ALS has been demonstrated by Vargas and coworkers who generated SOD1-G93A mice overexpressing Nrf2 specifically in astrocytes [122] and in neurons or type II skeletal muscle fibers [123]. In fact, Nrf2 overexpressing astrocytes isolated from SOD1-G93A transgenic mice could protect cocultured nontransgenic motor neurons from mutSOD1 toxicity by increasing the production/secretion of glutathione. Additionally, the overexpression of Nrf2 in astrocytes of SOD1-G93A transgenic mice increased the median survival in these animals [122]. Differently, Nrf2 overexpression in either neurons or type II skeletal muscle fibers in the same ALS mouse model could delay disease onset but could not extend life span [123]. A similar result has been reported by Guo and colleagues [124], who demonstrated that Nrf2 knockout in SOD1-G93A transgenic mice only modestly impacted the course of ALS. Taken together, these findings suggest that the antioxidant and prosurvival effects exerted by Nrf2 activation are important in modulating ALS phenotype but cell type specificity represents a critical factor to take into consideration when designing effective Nrf2-based pharmacological strategies for ALS treatment. 

## 6. Modulators of Nrf2/ARE Pathway

The Nrf2/ARE pathway can be pharmacologically activated by molecules of both natural derivation (nutraceuticals) and chemical synthesis. Sulforaphane (SFN), polyphenols, epigallocatechin 3-gallate (EGCG), and 1,7-bis(4-hydroxy-3-methoxyphenyl)-1,6-heptadiene-3,5-dione, alias curcumin, are among Nrf2/ARE activators of natural origin, whereas chemical Nrf2/ARE activators include triterpenoids and N-(4-(2-pyridyl)(1,3-thiazol-2-yl))-2-(2,4,6-trimethylphenoxy) acetamide, alias CPN-9.

A variety of nutraceuticals have demonstrated antioxidant and neuroprotective activity through Nrf2/ARE pathway induction. SFN, a naturally occurring isothiocyanate derived from cruciferous vegetables such as broccoli, activates Nrf2 via modification of reactive cysteine residues of Keap1 [102, 103], thereby providing protection in various models of neurodegeneration. In particular, it was shown that SFN is able to cross the blood brain barrier, activate Nrf2-dependent gene expression in the basal ganglia, and protect nigral dopaminergic neurons from cell death induced by MPTP [28]. Important Nrf2/ARE pathway activators are also EGCG and resveratrol, belonging to the family of polyphenols. Taking into account their abundance and multiple antioxidant mechanisms, polyphenols are considered to be important nutraceuticals. EGCG, a flavonoid polyphenol, is the main antioxidant molecule present in green tea. It displayed notable antioxidant and neuroprotective functions in cultured motoneuron-neuroblastoma hybrid cell line transfected with mutSOD1 [110] and in PC12 cells exposed to paraquat [111]. Furthermore, EGCG was shown to be neuroprotective in mice model of ALS: oral administration to mice expressing mutSOD1 delayed symptoms onset [112, 113]. EGCG was shown to activate Nrf2/ARE through protein kinase cascades [125]. Notably, it was demonstrated that EGCG activated HO-1 expression via Nrf2/ARE pathway, protecting rat neurons against oxidative insult [114]. Resveratrol, a polyphenolic compound present in red wine, demonstrated protective effects against hypoxic injury in rat spinal cord dorsal column by activating Nrf2 pathway [115]. Similarly, Nrf2 stabilization mediated by resveratrol protected dorsal root ganglion (DRG) neurons from glucose-induced injury [116]. Curcumin, a member of the curcuminoid family isolated from turmeric, the yellow rhizome of the plant *Curcuma longa*, showed Nrf2-dependent antioxidant properties in primary spinal cord astrocytes exposed to H_2_O_2_ [104] and in ischemic brain injury models [105]. Other natural activators of Nrf2/ARE pathway, such as naphthazarin, genistein, and carnosic acid, showed positive effects in several models of neurodegenerative and cardiovascular diseases implicating OS as a pathogenic factor [106–109, 126–128]. A summary of nutraceutical activators of Nrf2/ARE pathway described in this paragraph is reported in Table 1.

Together with molecules of natural origin, several synthetic Nrf2/ARE activators were recently developed. Notably, important obstacles in the identification of Nrf2/ARE chemical activators were encountered, mainly because of the highly time- and money-demanding conventional screening methods and the lack of structural similarities among developed molecules, thereby preventing coherent and convenient structure activity relationships (SAR) applications.

Recently, triterpenoids emerged as a potent class of Nrf2/ARE inducers. The triterpenoid family consists in three chemically related members: 2-cyano-3,12-dioxooleana-1,9-dien-28-oic acid ethylamide (CDDO-EA), CDDO trifluoroethylamide (CDDO-TFEA), and CDDO methylamide (CDDO-MA). Triterpenoids have shown a paramount potency in Nrf2 induction, the ability to attenuate dopaminergic neurodegeneration in MPTP mouse model of PD [117], and increase the life span in ALS mouse models [118]. Another interesting small chemical activator of Nrf2/ARE pathway is CPN-9 which selectively suppresses cell death triggered by OS in a cell-type-independent manner. SH-SY5Y cells pretreated with CPN-9 were more resistant to cytokine-induced apoptosis. CPN-9 also significantly suppressed ROS levels through the induction of several defensive genes [119]. Finally it is worth mentioning that some well-established therapeutics such as bromocriptine [120] and azathioprine [121] were reported to induce the Nrf2/ARE pathway, therefore providing insight into a possible development of new synthetic cutting edge Nrf2 activators. A summary of synthetic activators of Nrf2/ARE pathway described in this paragraph is reported in Table 2. 

## 7. Conclusion

OS is a hallmark of neurodegeneration and both PD and ALS report deregulated antioxidant defences and increased levels of OS markers in neural cells, biological fluids, and peripheral tissues.

Proteins such as DJ-1 and SOD1 are critical in PD and ALS pathogenesis and are also major players in the association between the neurodegenerative process and redox homeostasis. In fact, DJ-1 plays a protective role against OS and pathogenic mutations in *PARK7* gene lead to PD because of loss of this function. Differently, mutSOD1, through the gain of prooxidant and toxic function, aggregates and accumulates in the cell eventually causing ALS. According to recent studies, DJ-1 antioxidant capacity is not restricted to the protection of the neurons affected in PD but is rather a general function. Therefore, the activation of DJ-1 antioxidant downstream targets could be potentiated in order to obtain beneficial effects also in other neurodegenerative conditions. 

Several studies showed that both DJ-1 and SOD1 have a remarkable connection with the antioxidant Nrf2/ARE pathway, with DJ-1 being an upstream activator and SOD1 a target. As we explained in this review, the importance of Nrf2/ARE pathway lies in the ability to activate the expression of crucial antioxidant and detoxifying genes. Modulation of Nrf2/ARE pathway in neurodegenerative diseases, either with nutraceuticals or chemically synthesized molecules, might therefore augment cellular defences against OS, thus leading to neuroprotection [60].

In line with other studies, we conclude that pharmacological activation of Nrf2/ARE pathway represents an attractive neuroprotective therapy which may hold superior power as compared to conventional antioxidant routes and might represent a disease-modifying treatment to counteract neuronal loss in different degenerative pathologies.

## Figures and Tables

**Table 1:** Natural activators of Nrf2/ARE signalling pathway.

Molecule	Model	Reference
Sulforaphanes (SFN)	Keap-1 purified protein	Dinkova-Kostova et al., 2002 [102]
	Keap-1 protein (*in vitro *study)	Hong et al., 2005 [103]
	Dopaminergic neurons (basal ganglia) after exposure to MPTP	Jazwa et al. 2011 [28]

Curcumin	Spinal cord primary astrocytes after exposure to H_2_O_2_	Jiang et al., 2011 [104]
	Primary cortical neurons after oxygen-glucose deprivation/reoxygenation (model of brain ischemia)	Wu et al., 2013 [105]

Naphthazarin	ARE-bla Hep G2 cell line and primary neuron and astrocyte cultures	Son et al., 2013 [106]

Genistein	PC12 cells after incubation with beta-amyloid peptides 25–35	Ma et al., 2010 [127]
	bEND.3 cells after incubation with beta-amyloid peptides 25–35	Xi et al., 2012 [128]
	Rat hippocampal CA1 neurons after cerebral ischemia	Wang et al., 2013 [107]

Carnosic acid	Primary cortical neurons	Satoh et al., 2008 [108]
	SH-SY5Y cells after exposure to 6-hydroxydopamine	Chen et al., 2012 [109]

Polyphenols		
Epigallocatechin 3-gallate (EGCG)	mutSOD1-transfected motoneurons	Koh et al., 2004 [110]
	PC12 cells after exposure to paraquat	Hou et al., 2008 [111]
	SOD1-G93A transgenic mice (model of ALS)	Koh et al., 2006 [112]
	SOD1-G93A transgenic mice (model of ALS)	Xu et al., 2006 [113]
	Rat immortalized neurons (H19-7)	Romeo et al., 2009 [114]

Resveratrol	Spinal cord from adult rats after hypoxic injury	Kesherwani et al., 2013 [115]
	Dorsal root ganglionic cells after glucose-induced injury	Vincent et al., 2009 [116]

**Table 2:** Synthetic activators of Nrf2/ARE signalling pathway.

Molecule	Model	Reference
Triterpenoids		
CDDO-EA	MPTP-treated mice (model of PD)	Kaidery et al., 2013 [117]
CDDO-TFEA	Cellular models and SOD1-G93A transgenic mice (model of ALS)	Neymotin et al., 2011 [118]
CPN-9	Cellular models and SOD1^H46R ^transgenic mice (model of ALS)	Kanno et al., 2012 [119]
Bromocriptine	PC12 cells	Lim et al., 2008 [120]
Azathioprine	Cellular and transgenic mice (models of skin and liver carcinoma)	Kalra et al., 2011 [121]
